# Direct Metagenomic Detection of Viral Pathogens in Nasal and Fecal Specimens Using an Unbiased High-Throughput Sequencing Approach

**DOI:** 10.1371/journal.pone.0004219

**Published:** 2009-01-19

**Authors:** Shota Nakamura, Cheng-Song Yang, Naomi Sakon, Mayo Ueda, Takahiro Tougan, Akifumi Yamashita, Naohisa Goto, Kazuo Takahashi, Teruo Yasunaga, Kazuyoshi Ikuta, Tetsuya Mizutani, Yoshiko Okamoto, Michihira Tagami, Ryoji Morita, Norihiro Maeda, Jun Kawai, Yoshihide Hayashizaki, Yoshiyuki Nagai, Toshihiro Horii, Tetsuya Iida, Takaaki Nakaya

**Affiliations:** 1 Department of Genome Informatics, Research Institute for Microbial Diseases (RIMD), Osaka University, Suita, Osaka, Japan; 2 International Research Center for Infectious Diseases, Research Institute for Microbial Diseases (RIMD), Osaka University, Suita, Osaka, Japan; 3 Department of Virology, Research Institute for Microbial Diseases (RIMD), Osaka University, Suita, Osaka, Japan; 4 Department of Infectious Diseases, Osaka Prefectural Institute of Public Health, Higashinari, Osaka, Japan; 5 Department of Molecular Protozoology, Research Institute for Microbial Diseases (RIMD), Osaka University, Suita, Osaka, Japan; 6 Department of Virology 1, National Institute of Infectious Diseases, Musashimurayama, Tokyo, Japan; 7 Center of Research Network for Infectious Diseases, RIKEN, Chiyoda, Tokyo, Japan; 8 Omics Science Center (OSC), RIKEN, Yokohama, Kanagawa, Japan; Institut Pasteur Korea, Republic of Korea

## Abstract

With the severe acute respiratory syndrome epidemic of 2003 and renewed attention on avian influenza viral pandemics, new surveillance systems are needed for the earlier detection of emerging infectious diseases. We applied a “next-generation” parallel sequencing platform for viral detection in nasopharyngeal and fecal samples collected during seasonal influenza virus (Flu) infections and norovirus outbreaks from 2005 to 2007 in Osaka, Japan. Random RT-PCR was performed to amplify RNA extracted from 0.1–0.25 ml of nasopharyngeal aspirates (N = 3) and fecal specimens (N = 5), and more than 10 µg of cDNA was synthesized. Unbiased high-throughput sequencing of these 8 samples yielded 15,298–32,335 (average 24,738) reads in a single 7.5 h run. In nasopharyngeal samples, although whole genome analysis was not available because the majority (>90%) of reads were host genome–derived, 20–460 Flu-reads were detected, which was sufficient for subtype identification. In fecal samples, bacteria and host cells were removed by centrifugation, resulting in gain of 484–15,260 reads of norovirus sequence (78–98% of the whole genome was covered), except for one specimen that was under-detectable by RT-PCR. These results suggest that our unbiased high-throughput sequencing approach is useful for directly detecting pathogenic viruses without advance genetic information. Although its cost and technological availability make it unlikely that this system will very soon be the diagnostic standard worldwide, this system could be useful for the earlier discovery of novel emerging viruses and bioterrorism, which are difficult to detect with conventional procedures.

## Introduction

Acute respiratory infections and diarrhea are the leading causes of childhood morbidity and mortality worldwide, each resulting in an estimated nearly 2 million deaths annually [1], [2]. The diagnosis of respiratory and gastric/digestive infections is complex, due to the wide range of potential pathogens that can present with the same clinical symptoms [3]. In addition to the many known causes of these infections, it has been suggested that unrecognized infectious agents, including viruses, remain to be discovered [4]. It is estimated that, on average, up to 40% of diarrhea cases are of unknown etiology [4] and that the majority (69%) of upper respiratory infections are caused by viruses, including undiscovered ones [1].

Nucleic acid amplification tests (NATs) are increasingly being used for the diagnosis of viral infections. The most familiar formats use DNA or RNA target amplification methods, such as reverse transcription (RT) PCR, and have sensitivities that are greater than culture- or antigen-based procedures [3]. Loop-mediated isothermal amplification is more convenient and sensitive than PCR in amplifying DNA targets, and can be combined successfully with an RT step for RNA respiratory viruses. However, the wide variety of potential pathogens that elicit similar clinical symptoms and diseases makes the application of individual DNA- or RNA-based diagnostic assays both complex and expensive. Even multiplex PCRs are limited to 20–30 candidate pathogens, and may be confounded if viral evolution results in mutations at the primer binding sites [2]. DNA microarrays offer unprecedented opportunities for multiplexing; however, they are not widely implemented in clinical microbiology laboratories because of problems with sensitivity, throughput, and validation [2]. In addition, these microarrays are unavailable for unknown and/or unexpected microbes, as they require genetic information for each tested pathogen.

Newly-developed “next-generation” sequencing technologies, such as 454 (Roche), Solexa (Illumina), and SOLiD (ABI), allow researchers, in an unbiased manner, to obtain millions of sequences in a single round of operation [5]. Among these sequencing technologies, 454 currently offers by far the longest read length, ∼250 bp on the Genome Sequencer (GS) FLX platform [6]. Sequencing error levels are low (<1%) and arise primarily from homopolymer runs [7], but tend to be resolved in cases where there is sufficient coverage depth to allow the assembly of overlapping reads [8]. Many studies have used 454 pyrosequencing for the analysis of PCR amplicons, bacterial artificial chromosomes, genomic, mitochondrial, plastid DNA, and expression profiling [9], [10], [11], [12], [13], [14]. 454 is also a powerful tool for pathogen discovery [15], and was used with the GS platform to identify a new arenavirus transmitted through solid-organ transplantation [16] and a new polyomavirus in samples of Merkel cell skin carcinoma [17]. The 454 sequencing technique was also used to implicate Israeli acute paralysis virus as a significant marker for colony collapse disorder in honey bees [18]. Another group reported the whole genome analysis of Gallid herpesvirus, and showed that >99.0% coverage was obtained by assembling the raw sequence data to an overall average coverage depth of 13 [19].

We previously demonstrated the direct detection of a bacterial pathogen from a patient sample using 454 high-throughput DNA sequencing [20]. Here we report the design and diagnostic validation of an unbiased high-throughput sequencing method for the direct diagnosis of viral infections in clinical specimens. Patient samples were obtained during seasonal influenza virus (Flu) infections and norovirus outbreaks from 2005 to 2007 in Osaka, Japan. cDNAs, as templates for the GS FLX platform, were prepared by random RT-PCR using RNAs extracted from clinical samples. High-throughput sequencing yielded 15,298–32,335 sequences, of which 7–15,260 represented the targeted viral sequences. Furthermore, sequences of two recently identified human viruses, WU polyomavirus (WUV) and human coronavirus (HCoV) HKU1, were detected from the nasal and fecal samples, respectively.

## Results

### Random RT-PCR Amplification by the Transplex WTA Kit

**Figure 1:**
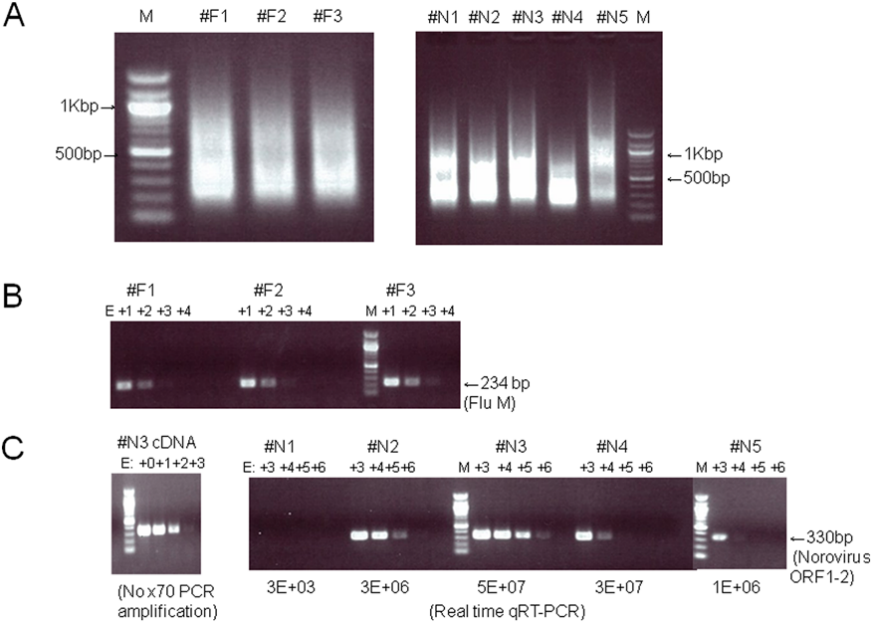
Random RT-PCR amplification of cDNA from clinical specimens and quantitative RT-PCR with virus-specific primers. The samples were nasopharyngeal aspirates and stools (n = 3 and 5, respectively) isolated during 2005–2007 in Osaka, Japan. Influenza A virus and norovirus were detected in the nasopharyngeal aspirates and stool samples, respectively, with other diagnostic methods. (A) RNA extracted from clinical specimens was reverse-transcribed and random-PCR amplified to prepare template DNA for pyrosequencing. One microgram of amplified PCR products in each sample was loaded onto a 1% agarose gel. M indicates 100-bp DNA ladder (NEB). (B) Flu-specific semi-quantitative PCR was performed with 10-fold serial dilutions of the random-PCR products. (C) Norovirus (GII)-specific semi-quantitative PCR was performed with 10-fold serial dilutions of the random-PCR products. Quantitative real-time RT-PCR using a norovirus-specific primer set was also performed, and the estimated copy numbers of norovirus in samples #N1–#N5 are shown in the panel on the right. As a control, cDNA from sample #N3 without random PCR amplification was used (left panel).

Total RNA isolated from either nasopharyngeal aspirates or fecal samples (0.1–0.2 ml) was under-measurable with an ND-1000 spectrophotometer (NanoDrop Technologies). Therefore, we performed quasi-random RT-PCR amplification using a whole transcriptome amplification (WTA) kit, according to the manufacturer's protocol with modifications, i.e. 70 cycles of PCR [21]. After random RT-PCR amplification, 10–13 µg of cDNA were obtained from the nasal and fecal samples (Figure 1A).

Semi-quantitative PCR was performed using 10-fold serial dilutions of the amplified cDNA as templates. Flu-specific PCR detected positive signals in all three nasopharyngeal aspirates (Figure 1B), and norovirus-specific primer sets detected the norovirus genome in four fecal samples, excluding #N1 sample (Figure 1C). The endpoint of detection in sample #N3 without 70 cycles of PCR amplification was E+03 (Figure 1C, left panel), whereas that with PCR amplification was E+06 (Figure 1C, right panel), suggesting that the viral cDNA was amplified almost 1,000 times by the 70 cycles of PCR. Together with the results that total RNA/cDNA were also amplified from several nanograms (data not shown) to ∼10 µg by random RT-PCR, these results indicate that viral genomes can be amplified similar to other DNAs with the WTA kit. Because almost all of the amplified cDNA were within the 200–1,000 bp range (Figure 1A), the PCR products were directly used as templates for emulsion PCR in the GS FLX pyrosequencing.

### Pyrosequencing Using the GS FLX Platform

**Figure 2:**
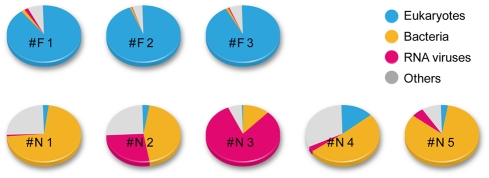
Pyrosequencing using the GS FLX system. Amplified cDNA was used as a template for GS FLX analysis. A 70×75 PicoTiterPlate device (gasket for 16 regions) was divided into 2 regions each for 8 samples. Obtained data were then subjected to a data analysis pipeline, as described in the Methods section. Comparison of the organisms from which the best matches for the sequences was shown.

The GS FLX system produces several million bases in one 7.5 h run [20]. The PicoTiterPlate device, physical divided into 16 regions, was used in this study, with 8 samples being loaded into 2 regions each. A single run yielded 15,298–32,335 (average 24,738) reads. Data analysis was basically performed according to the protocol we previously reported [20]. However, since the template cDNA for the high-throughput sequencing was prepared with random RT-PCR, an extra step was added to remove tag sequences in order to use the sequence data for BLASTN search analyses. Figure 2 shows the fraction of organisms (from which the sequences in the database were derived) that showed the best hits for the query sequences (E-value<10^−5^). To identify viral species including subtype, higher matches (E-value<10^−40^) for the sequence reads were selected (Table 1).

**Table 1:** Summary of detected viruses.

Sample	Age	Read	Virus
#F1	3	460	Influenza A virus (H3N2)
		3	Human endogenous retrovirus HCML-ARV
#F2	7	20	Influenza A virus (H1N1)
#F3	5	107	Influenza A virus (H3N2)
		7	WU Polyomavirus
#N1	62^a^	7	Norovirus (GII/4)
#N2	82^b^	7,304	Norovirus (GII/4)
#N3	92^b^	15,272	Norovirus (GII/4)
		813	Kyuri green mottle mosaic virus
		7	Citrus tristeza virus
		3	Enterobacteria phage phiK
#N4	3^c^	484	Norovirus (GII/4)
		14	Human coronavirus HKU1
		3	Phage phiV10
		3	Human endogenous retrovirus K
#N5	44^d^	762	Pepper mild mottle virus
		611	Norovirus (GII/4)
		17	Crucifer tobamovirus
		2	Tobacco mosaic virus

a Hospitalized patient.

b Patients in welfare facilities.

c Kindergarten student.

d Putative food.

### Nasal Samples

The Flu sequence was detected from all three samples in 21,858–30,958 (average 25,978) reads, as shown in Table 2. A partial genome was covered in these samples, and the cover rate ranged 8.1–58.3% (Table 2). One major reason for the partial coverage might be the large amount of host-derived sequences (90.0–94.6%; Table 2 and Figure 2), which were due to our direct RNA isolation from nasopharyngeal aspirates without the elimination of host or bacterial cells. However, 20–460 reads were Flu-derived, which was sufficient for subtype identification (H1N1 in sample #F2 and H3N2 in samples #F1 and #F3) from these sequences (Table S1).

**Table 2:** Summary of gene analysis in nasal samples.

Sample	#F1	#F2	#F3
**Total reads**	30,958 (100%)	25,119 (100%)	21,858 (100%)
**Eukaryotes**	27,849 (90.0%)	23,760 (94.6%)	20,296 (92.9%)
**Bacteria**	572 (1.85%)	230 (1.23%)	272 (1.24%)
**RNA viruses**	506 (1.63%)	21(0.10%)	121 (0.55%)
**Others**	2,031 (6.56%)	1,108 (1.63%)	1,169 (5.35%)
**Influenza A virus**	460 (1.49%)	20 (0.08%)	103 (0.49%)
Mapping to influenza A virus genome.			
**Cover rate**	58.30%	8.10%	25.60%
**Avg. depth**	3.67	0.23	0.65

The E value threshold was set to 1E+05 for taxonomy classification and 1E+40 for virus detection, respectively. Reference sequences used for mapping are CY026275-82, A/Texas/UR06-0566/2007(H3N2).

**Figure 3:**
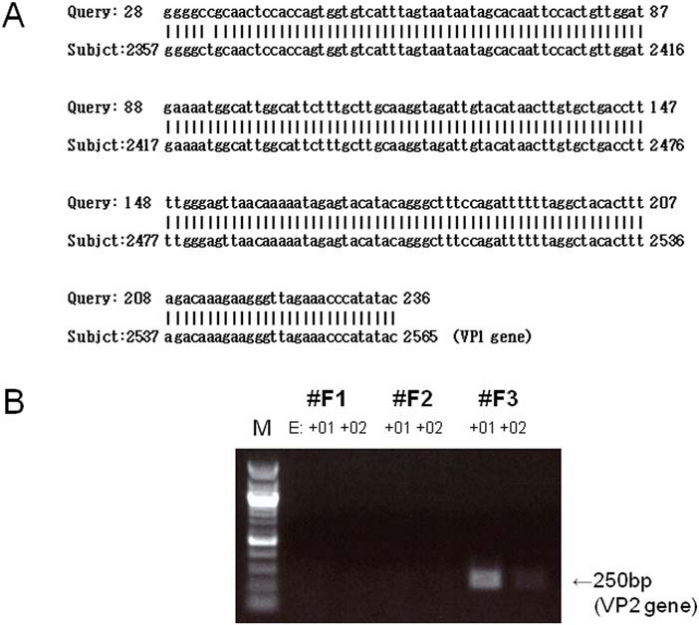
BLASTN (A) and PCR (B) analyses of WUV in Flu-positive nasopharyngeal aspirates. (A) Alignment of the WUV VP1 nucleotide sequence. The read obtained with the GS FLX sequencer (query) was compared with that of the WUV strain CLFF (subject; NCBI accession number: EU296475). (B) The WUV VP2 gene was detected by PCR using cDNA, which was amplified with random RT-PCR, as a template. The cDNA was diluted 10- and 100-fold and the PCR product was loaded on a 1% agarose gel. M indicates 100-bp DNA ladder.

In addition to the Flu sequence, a WUV-derived sequence was detected in one specimen (#F3) (Figure 3A). Because the detected sequences were located in a single gene (VP1), the presence of a second gene (VP2) was confirmed with PCR (Figure 3B). WUV and another novel human polyomavirus KI were cloned from respiratory tract specimens in 2007 [20], [22], [23], [24]. Although their etiological role in childhood respiratory disease has been proposed [23], [24], inconsistent epidemiological results have been reported [25]. In this study, the WUV-positive patient was a kindergarten student who was co-infected with Flu, consistent with the report by Norje et al. [25]. Partial sequences of the human endogenous retrovirus HCML-ARV were detected in sample #F1 (Table 1), although the pathogenesis of this virus is unknown.

The number of bacterial sequences read was 572 (#F1), 230 (#F2), and 272 (#F3), and the relative ratio to the total number of reads was 1.9%, 1.2%, and 1.2%, respectively (Table 2). The most abundantly detected bacterial sequences were *Streptococcus pneumoniae*, *Moraxella bovis*, *Moraxella bovoculi*, *Haemophilus influenzae*, and *Escherichia coli* (data not shown), which were present as major bacteria in the respiratory tract of children.

### Fecal Samples

To remove bacteria and human cells present in the feces, 15,000 rpm centrifugation was performed and the supernatants were used for RNA isolation. The norovirus sequence was detected from all five samples in 15,298–32,335 (average 23,994) reads, as summarized in Table 3. In contrast with influenza virus, almost the whole genome was covered in #N2 (7,302 reads) and #N3 (15,260 reads) samples, with average cover depths of 141.5 and 258.7, respectively (Table 3). More than 75% of the genome was covered in #N4 (484 reads) and #N5 (611 reads) samples (Table 3). A BLAST search of each sequence strongly indicated that these four patients were infected with a similar genotype, GII.4 (Table S2), consistent with previous diagnostic results [26]. In contrast, only 7 reads were detected in sample #N1 (Table 3), which was under-detectable with single round of PCR (Figure 1C), suggesting that the diagnostic method using high-throughput pyrosequencing is more sensitive than conventional PCR analysis.

**Table 3:** Summary of gene analysis in fecal samples.

Sample	#N1	#N2	#N3	#N4	#N5
**Total reads**	15,298 (100%)	32,335 (100%)	25,500 (100%)	18,014 (100%)	28,823 (100%)
**Eukaryotes**	400 (2.61%)	1,031 (22.7%)	147 (0.58%)	2,574 (14.3%)	948 (3.29%)
**Bacteria**	10,963 (71.7%)	14,423 (48.0%)	3,039 (11.9%)	9,180 (51.0%)	23,955 (83.1%)
**RNA viruses**	11 (0.07%)	8,742 (27.4%)	20,775 (81.5%)	546 (3.03%)	1,571 (5.45%)
**Others**	3,924 (25.7%)	8,139 (25.2%)	1,539 (6.04%)	5,714 (31.7%)	2,349 (8.15%)
**Norovirus**	7 (0.05%)	7,302(22.6%)	15,260 (59.8%)	484 (2.69%)	611 (2.12%)
Mapping to Norovirus genome					
**Cover rate**	2.10%	97.00%	98.00%	77.50%	84.50%
**Avg. depth**	0	141.5	258.7	9.3	12.5

Reference sequence used for mapping is AY587989, Norovirus Hu/NLV/Oxford/B2S16/2002/UK.

One-step real time RT-PCR (qRT-PCR) was also performed on the extracted RNA. The estimated copy number of norovirus in each fecal sample is shown in Figure 1C. The copy number of norovirus in sample #N1 with qRT-PCR was 3E+03, whereas those of other samples ranged from 1E+06 to 5E+07. The relative copy numbers of norovirus in samples #N1 to #N3, which were isolated in 2006 (October to December), were almost consistent with the semi-quantitative PCR results, although the sensitivity of the semi-quantitative PCR was 10-fold lower than that of qRT-PCR (Figure 1C). By contrast, the real-time RT-PCR and semi-quantitative PCR were ∼1,000-fold different in samples #N4 and #N5 (Figure 1C), which were isolated in May 2005 and January 2006, respectively. In these samples, RNA isolation for 454 pyrosequencing was performed in February 2007, whereas RNA for qRT-PCR was isolated just after sample collection. Thus, the above inconsistencies might be due to the storage periods of the samples (at 4°C).

HCoV-HKU1, which was recently identified as the fifth human coronavirus [27], was detected in one specimen (#N4). A total of 14 reads from 18,014 reads matched to the HCoV-HKU1 virus, with four regions being detected (Table 4). Epidemiological studies have reported that HCoV-HKU1 was found in the nasopharyngeal aspirates of 10/418 (2.4%) studied patients with community-acquired pneumonia [28], and that HCoV-HKU1 could be detected in respiratory and stool samples from children and adults. Studies have also reported a 9-month-old patient who was co-infected with HCoV-HKU1 and influenza C virus [29]. We showed here that a 5-year-old child was co-infected with HCoV-HKU1 and norovirus, although the relationship between these two viruses and/or the relationship between pathogenesis (enteric tract illness) and co-infection of these two viruses remains unknown. Other human coronaviruses (OC-43, 229E, and NL63) were not detected from these fecal samples or from the nasal samples with RT-PCR (data not shown). Human endogenous retrovirus K (HERV-K)-derived sequences was also detected in patient #N4, who was 3 years old (Table 1). HERV-K is the name given to an approximately 30-million-year-old family of endogenous retroviruses present at >50 copies per haploid human genome [30].

**Table 4:** Detected regions in HCoV-HKU1 genome.

Region (nt)	Genome
3,854–4,083	Orf 1ab (Replicase)
15,956–16,147	Orf 1ab (Replicase)
24,506–24,653	Spike glycoprotein
28,082–28,310	Membrane glycoprotein - Nucleocapsid phosphoprotein

Reference sequence: HCoV HKU1 strain N15 genotype B, complete genome (NCBI accession number: DQ415911).

In addition to these human viruses, several plant virus–derived sequences were also detected in the fecal samples (Table 1). In particular, pepper mild mottle virus (PMMV) was found in two specimens (#N1 and #N5) and was confirmed with RT-PCR (data not shown). The total number of PMMV reads outnumbered the norovirus reads in sample #N5 (Table 1). In addition, Kyuri green mottle mosaic virus (KGMMV) was also abundantly detected in sample #N3 (813 KGMMV-specific reads in a total 25,500 reads). KGMMV was also detected after ultracentrifugation (data not shown), suggesting that KGMMV viral particles were present in the human gut. Previously, PMMV was detected at 10^9^ virions per gram of dry weight fecal matter, and was detected in 12 (66.7%) of the 18 fecal samples collected from healthy humans [31]. This previous publication also showed that fecal PMMV was infectious to host plants [31]. Therefore, these plant-derived viruses may retain their infectivity in the feces and may even be present in diarrhea. Almost all of the detected viruses, except for the citrus tristeza virus, a member of the *Closterovirus* group, belong to the *Tobamovirus* group (Table 1). It is currently unknown if there is an interaction between these plus-stranded RNA viruses and norovirus. The most abundantly detected PMMV was also found in healthy humans [31]. Although the previous paper [31] reported that there is a lack of evidence to show that active replication of PMMV occurs in human feces, further investigations regarding plant virus replication in the human gut (epithelial cells) seem necessary.

As shown in Table 3, 3,039–23,955 (11.9–83.1%) reads were estimated to be bacterial genes. Of those, more than half (54.7–69.9%) were rRNA-derived (Table 5) and a BLAST searching predicted the existence of commensal bacteria in the human intestine (data not shown).

**Table 5:** Summary of bacterial and human-derived gene analysis.

Sample	#F1	#F2	#F3
**Total reads**	30,958	25,119	21,858
**Human**	26,957 (100%)	23,029 (100%)	19,612 (100%)
rRNA	252 (0.93%)	31 (0.13%)	203 (1.04%)
coding region^a^	637 (2.36%)	298 (1.29%)	449 (2.29%)
non-coding region^b^	21,208 (78.7%)	18,035 (78.3%)	15,226 (77.6%)

Sample	#N1	#N2	#N3	#N4	#N5
**Total reads**	15,298	32,335	25,500	18,014	28,823
**Bacteria**	10,963 (100%)	14,423 (100%)	3,039 (100%)	9,180 (100%)	23,955 (100%)
rRNA	5,994 (54.7%)	10,076 (69.9%)	1,829 (60.2%)	5,424 (59.1%)	13,772 (57.5%)

a Hit reads with exon region.

b Hit reads with intron and intergenic regions.

## Discussion

**Figure 4:**
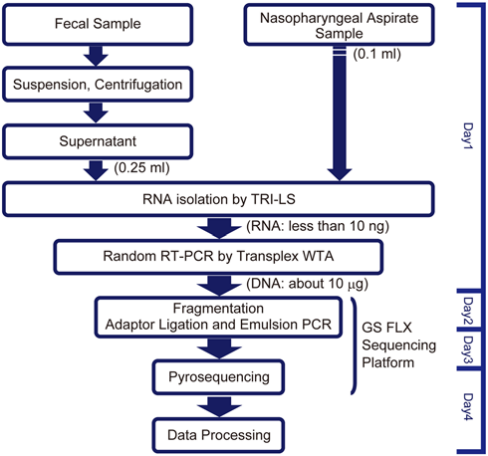
Process diagram for the viral diagnosis of nasopharyngeal aspirates and fecal samples.

In this study, we demonstrated the potential of the 454 parallel sequencing platform to identify pathogenic viruses from clinical specimens. We chose random RT-PCR for template cDNA preparation, because of the low levels of isolated RNA from the specimens. Flu and norovirus were detected from all three nasopharyngeal aspirates and five stool specimens, respectively, consistent with other diagnostic methods, including RT-PCR. In addition to these viruses, possible pathogenic viruses, such as WUV and HCoV-HKU1, were also detected in the nasopharyngeal aspirates and fecal samples, respectively, suggesting that this system (Figure 4) is useful for novel virus identification as well as for viral genome analysis.

In the severe acute respiratory syndrome (SARS)-CoV outbreak in 2003, it was demonstrated that a combination of stool, pooled nasal, and throat swab specimens gave the highest yield for SARS-CoV detection by RT-PCR [32]. Thus, not only respiratory specimens but also gastric and digestive specimens are important for the diagnosis of emerging infectious viruses, including those with airborne transmission. In this study, we isolated whole RNA and detected viral genes from nasal and stool samples with the 454 high-throughput sequencing system. Flu sequences were present in 20–460 of the 21,858–30,958 reads in each nasopharyngeal aspirate (Table 1), and the cover rates ranged from 8.1–58.3% (Table 2), which was sufficient for subtype identification in all three specimens. Furthermore, the near-complete norovirus genome sequence was obtained in two fecal specimens (#N2 and #N3), and more than 75% of the genome was covered in the other two specimens (#N4 and #N5) (Table 3).

Recently, we subjected stool sample–extracted DNAs to 454 pyrosequencing, and found that nearly 20% of the reads had best hits that matched currently-reported bacterial DNA sequences [20]. These previous results, together with our findings here, indicate that two protocols, namely direct DNA extraction for bacteria and cell/bacterial removal by centrifugation followed by RNA/DNA extraction for virus, could be used to comprehensively identify pathogenic microbes in clinical samples.

Our preliminary experiments demonstrated that the detected number of viral sequences paralleled the virus copy number in blood samples (unpublished data), suggesting that this system is highly quantitative. Indeed, the copy number of norovirus in the #N1 to #N5 samples (Figure 1C), as measured by semi-quantitative PCR, was significantly correlated with the number of norovirus genome sequences as detected by high-throughput sequencing (Table 1). In the case of the nasal samples, the copy number of the influenza virus in sample #F2 was lower than those in samples #F1 and #F3, although the endpoints of the semi-quantitative PCR for these samples were comparable (Figure 1B). This inconsistency could be due to differences in the sensitivities of the primers used for H1N1 and H3N2 influenza viruses (Table 1) or to the presence of different amounts of host-derived DNA/cDNA. Thus, quantitative analysis of host genes will be required. One potential reason for why we obtained fewer Flu-specific reads than norovirus reads in this study might have been the large number (90.0–94.6% of all reads) of host-derived sequences (Figure 2 and Table 5). These sequences were present because we performed direct RNA isolation from nasopharyngeal aspirates without first eliminating the cells or tissues. Most of the detected human-derived reads were non-coding regions, and fewer coding regions, including rRNA and mRNA sequences, were detected than expected (Table 5). These results suggest that, although contamination by human genomic DNA might be very low, an additional step for host gene removal is required. Suitable subtraction step(s) using pooled human genomic DNAs as drivers might be required to enrich in microbial genomes [15]. Alternatively, MICROBEnrich (Ambion Inc.), another method for removing contaminated human-derived RNA, could be useful to enrich microbial RNA [33]. However, the DNA virus WUV was detected from the isolated RNA, suggesting that the WUV genome and/or its transcripts present in infected cells were detected. Indeed, a novel human polyomavirus (Merkel cell polyomavirus), isolated from skin carcinoma, was detected from mRNA [17]. Taken together, these results indicate that whole RNA isolation, including host cells and tissues, followed by the suitable elimination of host-derived genes could be an effective method for identifying pathogenic viruses in clinical samples.

When several pathogens are found in a single sample, a careful interpretation is necessary to decide which pathogen(s) is the real cause of a specific disease. Although, the most abundant pathogen might generally be considered to be the best candidate, cooperative interactions between multiple pathogens cannot be excluded as an important factor for pathogenesis. To address this question, suitable control samples from healthy persons and/or pair specimens, isolated after recovery, might be required.

Another possible problem with this viral genome analysis is biased cDNA synthesis by quasi-random RT-PCR with the WTA kit. As shown in Figure S1, a significant bias was found and its pattern was identical in all samples. TG (CA)–rich regions were selectively amplified with the WTA kit (Table S3), probably due to nucleotide sequences of the quasi-random primer. Random RT-PCR amplification using the WTA kit was at least one log higher than that using the conventional random hexamer (data not shown). This suggests that further improvement is required for whole viral genome analysis, although our system is suitable for the comprehensive detection of viral genes. In addition, the TG (CA)–rich bias was observed within the viral genome; therefore, it seems unlikely that the bias leads to quantitative differences of the detected sequences with respect to the original population.

Almost all diagnostic NATs require viral genome information, and thus cannot be performed for novel or unexpected viral infections. In this study, we showed that a diagnostic system based on parallel high-throughput sequencing is useful for the direct detection of unknown and/or small numbers of viruses, as well as for the genetic characterization of major pathogenic viruses in clinical specimens. We plan to share this system domestically as well as with the Asian epidemic network (The Program of Founding Research Centers for Emerging and Reemerging Infectious Diseases; http://www.crnid.riken.jp), in order to enable the earlier identification of unknown pathogens in a novel outbreak or bioterrorism.

The cost of this approach will be a key concern for its adaption by the research community. Microbe-derived DNA/RNA enrichment [33], with suitable elimination of host-derived genes as described above, could reduce the required number of reads per sample. In addition, parallel tagged sequencing [6] using sample-specific barcoding adaptors with 5′-nucleotide tagged PCR primers [34] would enable the analysis of multiple samples in a single sequencing region. If these methods were combined, it would lead to significant reductions the operating costs (i.e., $2,000 per sample) of 90% or more.

This system, which can produce >0.4 million clones per run within a half-day, could also be very useful for the rapid identification of important mutation(s) by direct comparison with wild and mutant viruses, including “pandemic Flu” [35] and more virulent noroviruses [36], [37].

## Materials and Methods

### RNA isolation from clinical samples

We analyzed unlinked, anonymous samples in the Osaka Prefectural Institute of Public Health. The samples were nasopharyngeal aspirates and stools (n = 3 and 5, respectively) isolated during 2005–2007 in Osaka, Japan. Seasonal influenza A virus (Flu) in nasopharyngeal aspirates from 3- to 7-year-old children was detected by a rapid diagnostic kit (immunochromatography) using Flu-specific antibodies. In 2006/2007, a large-scale norovirus outbreak occurred in Osaka, Japan, mainly infecting patients in nursing homes and welfare facilities (53%), hospitals (27%), kindergartens (15%), and elementary and junior high schools (5%) [26]. #N1 to #N3 samples were collected during this outbreak. #N1 sample was derived from a hospitalized patient and #N2 and #N3 samples were derived from patients in a welfare facility in 2006 (October to December). #N4 sample was a kindergarten student when an outbreak occurred at the elementary school of its elder brother and sister. In contrast to these four cases of putative human-to-human transmission, #N5 sample was oyster-associated. #N4 and #N5 samples were collected in May 2005 and January 2006, respectively. Diagnosis of norovirus infection was based on RT–PCR [26]. The collected stool was suspended with an equal amount of PBS and was centrifuged at 15,000 rpm for 10 min. The supernatants (0.25 ml) were used for RNA isolation. This study was approved by the ethical review committees of the RIMD, Osaka University, Osaka Prefectural Institute of Public Health, National Institute of Infectious Diseases, and RIKEN.

### Quantitative RT-PCR of norovirus

RNA extraction was performed using a Magtration-MagaZorbRNA Common kit (Precision System Science) and the viral copy number of norovirus was estimated with One-step real time RT-PCR [26] using a One-Step Realtime PCR reagent kit (Toyobo). A plasmid containing the target sequence was used as a control.

### Random RT-PCR amplification

Total RNA was extracted from specimens with TRI-LS (Sigma-Aldrich), and was reverse-transcribed with the Transplex whole transcriptome amplification (WTA) kit (Sigma-Aldrich) [21] using a quasi-random primer, according to the manufacturer's protocol. PCR amplification for the preparation of template DNA for pyrosequencing was carried out by AmpliTaq Gold DNA Polymerase LD (Applied Biosystems) [21]. Norovirus-specific PCR was performed as described above [26], and Flu-specific PCR was performed using the FluA M gene-specific primer set (M30F: 5′-TTCTAACCGAGGTCGAAACG-3′ and M264R2: 5′-ACAAAGCGTCTACGCTGCAG-3′).

### RT- PCR diagnosis

Viral RNA was extracted from nasopharyngeal and fecal specimens with a QIAamp Viral RNA Mini Kit (QIAGEN), and cDNA was synthesized using SuperScript™ III reverse transcriptase (Invitrogen) with a random hexamer, as described previously [21]. The generated cDNA was subjected to PCR using the Expand High Fidelity^PLUS^ PCR System (Roche) with primer sets specific to viruses, such as human coronaviruses [38], WU polyomavirus [24], and PMMV [31].

### Pyrosequencing and data analysis

The amplified cDNA was used as a template for GS FLX analysis (454 Life Sciences). A 70×75 PicoTiterPlate device (gasket for 16 regions) was divided into 2 regions for each of 8 samples. The obtained data were then subjected to a data analysis pipeline. Data analysis was performed on each read sequence by computational tools, as constructed previously [20] with some modifications. The analysis steps were: (i) remove tag sequences; (ii) execute a BLASTN search by Hi-per BLAST (Fujitsu); (iii) identify the scientific name for each read based on the NCBI taxonomy database; (iv) extract viral reads and perform mapping to reference data by SSEARCH. This analysis pipeline was constructed by utilizing BioRuby [39], BioPerl [40], and MySQL. After classification, particular human and bacterial reads were further analyzed as follows. Human genome mapping was performed by MEGABLAST search against the Human Genome, Homo_sapiens.NCBI36.49, using a threshold of 1E-40. Bacterial rRNA typing was performed by BLASTN search against the comprehensive rRNA database “silva” release 94 [41] using a threshold of 80% match per read.

## Supporting Information

Figure S1 Cover depth of norovirus. Norovirus Hu/NLV/Oxford/B2S16/2002/UK (NCBI accession number: AY587989) was used as a reference sequence.
(0.21 MB PDF)

Table S1 Summary of the best hits for each query sequences (E-value<1E-40) in nasopharyngeal aspirates
(0.15 MB PDF)

Table S2 Summary of the best hits for each query sequences (E-value<1E-40) in fecal samples
(0.20 MB PDF)

Table S3 Bias towards TG(CA)-enrichment within the detected norovirus sequences in fecal samples
(0.10 MB PDF)

## References

[1] Lodes MJ, Suciu D, Wilmoth JL, Ross M, Munro S (2007). Identification of upper respiratory tract pathogens using electrochemical detection on an oligonucleotide microarray.. PLoS ONE.

[2] Quan PL, Palacios G, Jabado OJ, Conlan S, Hirschberg DL (2007). Detection of respiratory viruses and subtype identification of influenza A viruses by GreeneChipResp oligonucleotide microarray.. J Clin Microbiol.

[3] Fox JD (2007). Nucleic acid amplification tests for detection of respiratory viruses.. J Clin Virol.

[4] Finkbeiner SR, Allred AF, Tarr PI, Klein EJ, Kirkwood CD (2008). Metagenomic analysis of human diarrhea: viral detection and discovery.. PLoS Pathog.

[5] Fan HC, Blumenfeld YJ, Chitkara U, Hudgins L, Quake SR (2008). Noninvasive diagnosis of fetal aneuploidy by shotgun sequencing DNA from maternal blood.. Proc Natl Acad Sci U S A.

[6] Meyer M, Stenzel U, Hofreiter M (2008). Parallel tagged sequencing on the 454 platform.. Nat Protoc.

[7] Margulies M, Egholm M, Altman WE, Attiya S, Bader JS (2005). Genome sequencing in microfabricated high-density picolitre reactors.. Nature.

[8] Vera JC, Wheat CW, Fescemyer HW, Frilander MJ, Crawford DL (2008). Rapid transcriptome characterization for a nonmodel organism using 454 pyrosequencing.. Mol Ecol.

[9] Bainbridge MN, Warren RL, He A, Bilenky M, Robertson AG (2007). THOR: targeted high-throughput ortholog reconstructor.. Bioinformatics.

[10] Goldberg SM, Johnson J, Busam D, Feldblyum T, Ferriera S (2006). A Sanger/pyrosequencing hybrid approach for the generation of high-quality draft assemblies of marine microbial genomes.. Proc Natl Acad Sci U S A.

[11] Moore MJ, Dhingra A, Soltis PS, Shaw R, Farmerie WG (2006). Rapid and accurate pyrosequencing of angiosperm plastid genomes.. BMC Plant Biol.

[12] Poinar HN, Schwarz C, Qi J, Shapiro B, Macphee RD (2006). Metagenomics to paleogenomics: large-scale sequencing of mammoth DNA.. Science.

[13] Torres TT, Metta M, Ottenwälder B, Schlötterer C (2008). Gene expression profiling by massively parallel sequencing.. Genome Res.

[14] Wicker T, Schlagenhauf E, Graner A, Close TJ, Keller B (2006). 454 sequencing put to the test using the complex genome of barley.. BMC Genomics.

[15] MacConaill L, Meyerson M (2008). Adding pathogens by genomic subtraction.. Nat Genet.

[16] Palacios G, Druce J, Du L, Tran T, Birch C (2008). A new arenavirus in a cluster of fatal transplant-associated diseases.. N Engl J Med.

[17] Feng H, Shuda M, Chang Y, Moore PS (2008). Clonal integration of a polyomavirus in human Merkel cell carcinoma.. Science.

[18] Cox-Foster DL, Conlan S, Holmes EC, Palacios G, Evans JD (2007). A metagenomic survey of microbes in honey bee colony collapse disorder.. Science.

[19] Spatz SJ, Rue CA (2008). Sequence determination of a mildly virulent strain (CU-2) of Gallid herpesvirus type 2 using 454 pyrosequencing.. Virus Genes.

[20] Nakamura S, Maeda N, Miron IM, Yoh M, Izutsu K (2008). Metagenomic diagnosis of bacterial infections.. Emerg Infect Dis.

[21] Watanabe S, Mizutani T, Sakai K, Kato K, Tohya Y (2008). Ligation-mediated amplification for effective rapid determination of viral RNA sequences (RDV).. J Clin Virol.

[22] Allander T, Andreasson K, Gupta S, Bjerkner A, Bogdanovic G (2007). Identification of a third human polyomavirus.. J Virol.

[23] Bialasiewicz S, Whiley DM, Lambert SB, Wang D, Nissen MD (2007). A newly reported human polyomavirus, KI virus, is present in the respiratory tract of Australian children.. J Clin Virol.

[24] Gaynor AM, Nissen MD, Whiley DM, Mackay IM, Lambert SB (2007). Identification of a novel polyomavirus from patients with acute respiratory tract infections.. PLoS Pathog.

[25] Norja P, Ubillos I, Templeton K, Simmonds P (2007). No evidence for an association between infections with WU and KI polyomaviruses and respiratory disease.. J Clin Virol.

[26] Sakon N, Yamazaki K, Yoda T, Tsukamoto T, Kase T (2007). Norovirus storm in Osaka, Japan, last winter (2006/2007).. Jpn J Infect Dis.

[27] Woo PC, Lau SK, Chu CM, Chan KH, Tsoi HW (2005). Characterization and complete genome sequence of a novel coronavirus, coronavirus HKU1, from patients with pneumonia.. J Virol.

[28] Woo PC, Lau SK, Tsoi HW, Huang Y, Poon RW (2005). Clinical and molecular epidemiological features of coronavirus HKU1-associated community-acquired pneumonia.. J Infect Dis.

[29] Vabret A, Dina J, Gouarin S, Petitjean J, Corbet S (2006). Detection of the new human coronavirus HKU1: a report of 6 cases.. Clin Infect Dis.

[30] Yang J, Bogerd H, Le SY, Cullen BR (2000). The human endogenous retrovirus K Rev response element coincides with a predicted RNA folding region.. RNA.

[31] Zhang T, Breitbart M, Lee WH, Run JQ, Wei CL (2006). RNA viral community in human feces: prevalence of plant pathogenic viruses.. PLoS Biol.

[32] Chan PK, To WK, Ng KC, Lam RK, Ng TK (2004). Laboratory diagnosis of SARS.. Emerg Infect Dis.

[33] Lin B, Wang Z, Vora GJ, Thornton JA, Schnur JM (2006). Broad-spectrum respiratory tract pathogen identification using resequencing DNA microarrays.. Genome Res.

[34] Binladen J, Gilbert MT, Bollback JP, Panitz F, Bendixen C (2007). The use of coded PCR primers enables high-throughput sequencing of multiple homolog amplification products by 454 parallel sequencing.. PLoS ONE.

[35] Ilyushina NA, Govorkova EA, Gray TE, Bovin NV, Webster RG (2008). Human-like receptor specificity does not affect the neuraminidase-inhibitor susceptibility of H5N1 influenza viruses.. PLoS Pathog.

[36] Lopman B, Zambon M, Brown DW (2008). The evolution of norovirus, the “gastric flu”.. PLoS Med.

[37] Lindesmith LC, Donaldson EF, Lobue AD, Cannon JL, Zheng DP (2008). Mechanisms of GII.4 norovirus persistence in human populations.. PLoS Med.

[38] Lau SK, Woo PC, Yip CC, Tse H, Tsoi HW (2006). Coronavirus HKU1 and other coronavirus infections in Hong Kong.. J Clin Microbiol.

[39] Goto N, Nakao MC, Kawashima S, Katayama T, Kanehisa M (2003). BioRuby: Open-Source Bioinformatics Library.. Genome Informatics.

[40] Stajich JE, Block D, Boulez K, Brenner SE, Chervitz SA (2002). The Bioperl toolkit: Perl modules for the life sciences.. Genome Res.

[41] Pruesse E, Quast C, Knittel K, Fuchs BM, Ludwig W (2007). SILVA: a comprehensive online resource for quality checked and aligned ribosomal RNA sequence data compatible with ARB.. Nucleic Acids Res.

