# Supplementary figures and images for: Direct Metagenomic Detection of Viral Pathogens in Nasal and Fecal Specimens Using an Unbiased High-Throughput Sequencing Approach
Source: PLoS One. 2009 Jan 19;4(1):e4219. doi: 10.1371/journal.pone.0004219 (PMC2625441; doi:10.1371/journal.pone.0004219)

Figure S1.

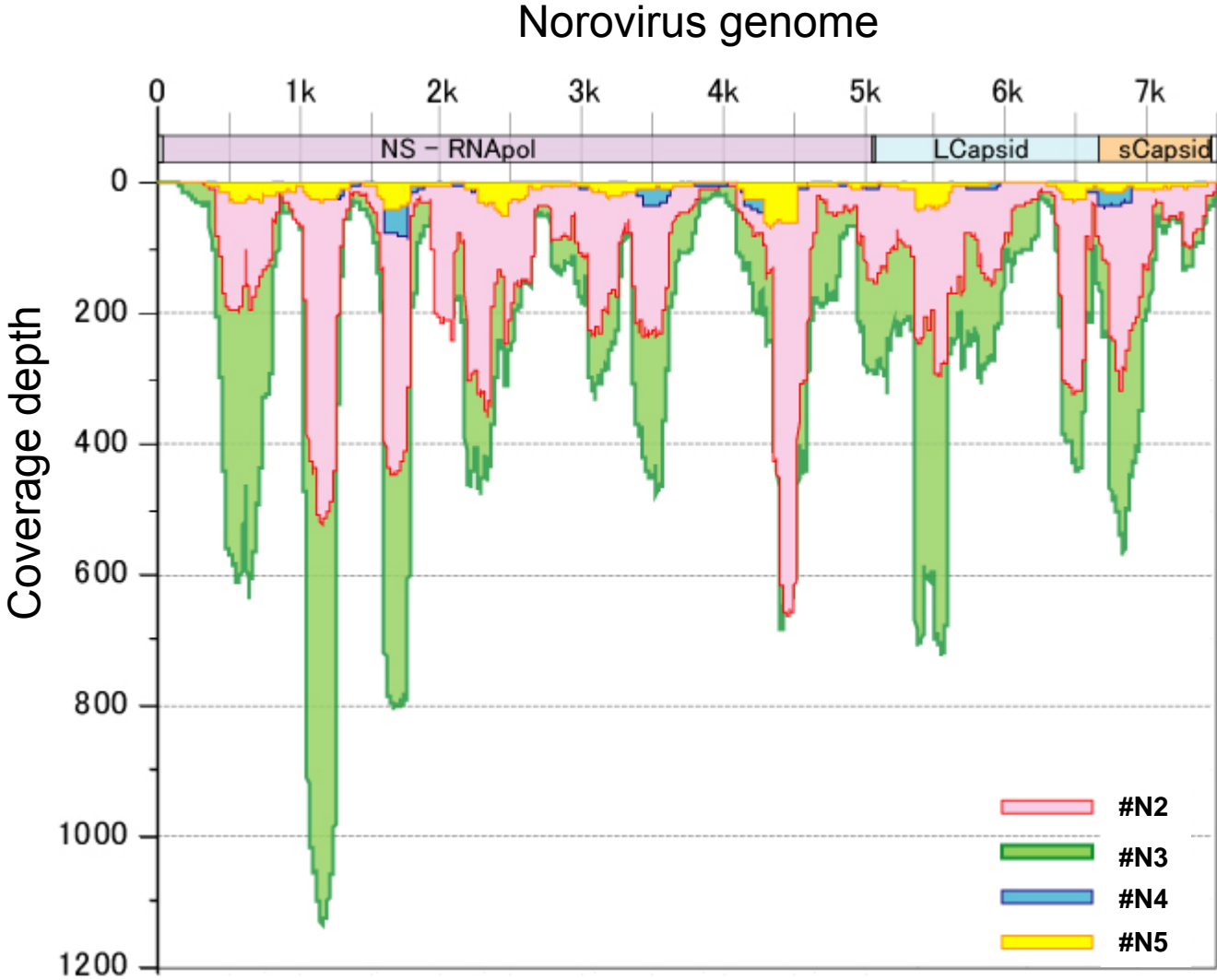

Supplement: Figure S1 — Cover depth of norovirus. Norovirus Hu/NLV/Oxford/B2S16/2002/UK (NCBI accession number: AY587989) was used as a reference sequence. (0.21 MB PDF) [file pone.0004219.s001.pdf]
