# Supplementary material for: Direct Metagenomic Detection of Viral Pathogens in Nasal and Fecal Specimens Using an Unbiased High-Throughput Sequencing Approach
Source: PLoS One. 2009 Jan 19;4(1):e4219. doi: 10.1371/journal.pone.0004219 (PMC2625441; doi:10.1371/journal.pone.0004219)
Supplement: Table S1 — Summary of the best hits for each query sequences (E-value<1E-40) in nasopharyngeal aspirates (0.15 MB PDF) [file pone.0004219.s002.pdf]

Table S1. Summary of the best hits for each query sequences (E-value < 10^{-40}) in nasopharyngeal aspirates

#F1			
Read	Virus	Read	Virus
55	Influenza A virus (A/Texas/UR06-0566/2007(H3N2))	2	Influenza A virus (A/New York/283/1999(H3N2))
52	Influenza A virus (A/Kentucky/UR06-0370/2007(H3N2))	2	Influenza A virus (A/New York/238/2005(H3N2))
43	Influenza A virus (A/Western Australia/69/2005(H3N2))	2	Influenza A virus (A/New York/214/2003(H3N2))
39	Influenza A virus (A/Canterbury/26/2005(H3N2))	2	Influenza A virus (A/Miyagi/S707/2006(H3N2))
31	Influenza A virus (A/Canterbury/205/2005(H3N2))	2	Influenza A virus (A/Miyagi/N1220/2005(H3N2))
29	Influenza A virus (A/New York/938/2006(H3N2))	2	Influenza A virus (A/Memphis/5/1988(H3N2))
26	Influenza A virus (A/Virginia/UR06-0580/2007(H3N2))	2	Influenza A virus (A/Canterbury/127/2005(H3N2))
11	Influenza A virus (A/South Australia/42/2005(H3N2))	2	Influenza A virus (A/California/8/2006(H3N2))
10	Influenza A virus (A/Virginia/UR06-0021/2006(H3N2))		
10	Influenza A virus (A/Queensland/52/2005(H3N2))		
10	Influenza A virus (A/Aichi/122/2006(H3N2))		
8	Influenza A virus (A/Oregon/UR06-0389/2007(H3N2))		
7	Influenza A virus (A/TW/875/04(H3N2))		
7	Influenza A virus (A/New South Wales/7/1999(H3N2))		
6	Influenza A virus (A/Washington/UR06-0252/2007(H3N2))		
6	Influenza A virus (A/Washington/UR06-0225/2007(H3N2))		
6	Influenza A virus (A/Sachsen-Anhalt/19/99(H3N2))		
6	Influenza A virus (A/New York/923/2006(H3N2))		
6	Influenza A virus (A/Miyagi/S736/2006(H3N2))		
5	Influenza A virus (A/USA/AF1086/2007(H3N2))		
5	Influenza A virus (A/New York/UR06-0515/2007(H3N2))		
5	Influenza A virus (A/New York/928/2006(H3N2))		
5	Influenza A virus (A/Aichi/82/2006(H3N2))		
4	Influenza A virus (A/Oregon/UR06-0200/2007(H3N2))		
4	Influenza A virus (A/Canterbury/258/2005(H3N2))		
3	Influenza A virus (A/El Salvador/AF1042/2006(H3N2))		
2	Influenza A virus (A/New York/933/2006(H3N2))		
2	Influenza A virus (A/New York/608/1996(H3N2))		
2	Influenza A virus (A/New York/485/2003(H3N2))		
2	Influenza A virus (A/New York/377/2004(H3N2))		
2	Influenza A virus (A/New York/365/2004(H3N2))		

Table S1. Summary of the best hits for each query sequences (E-value < 10^{-40}) in nasopharyngeal aspirates (continue)

#F2		#F3	
Read	Virus	Read	Virus
12	Influenza A virus (A/Waikato/13/2005(H1N1))	20	Influenza A virus (A/Thailand/426/2005(H3))
3	Influenza A virus (A/Virginia/UR06-0295/2007(H1N1))	12	Influenza A virus (A/Canterbury/16/2005(H3N2))
		11	Influenza A virus (A/South Australia/20/2005(H3N2))
		10	Influenza A virus (A/Kentucky/UR06-0370/2007(H3N2))
		**7**	**WU Polyomavirus**
		7	Influenza A virus (A/New York/908/2004(H3N2))
		5	Influenza A virus (A/Wellington/4/2005(H3N2))
		5	Influenza A virus (A/Waikato/12/2005(H3N2))
		4	Influenza A virus (A/Egypt/96/2002(H1N2))
		4	Influenza A virus (A/Christchurch/10/2004(H3N2))
		3	Influenza A virus (A/New York/913/2005(H3N2))
		2	Influenza A virus (A/Western Australia/65/2005(H3N2))
		2	Influenza A virus (A/Waikato/1/2004(H3N2))
		2	Influenza A virus (A/Canterbury/127/2005(H3N2))
		2	Influenza A virus (A/Brisbane/1/2006(H3))
