# Supplementary material for: Direct Metagenomic Detection of Viral Pathogens in Nasal and Fecal Specimens Using an Unbiased High-Throughput Sequencing Approach
Source: PLoS One. 2009 Jan 19;4(1):e4219. doi: 10.1371/journal.pone.0004219 (PMC2625441; doi:10.1371/journal.pone.0004219)
Supplement: Table S2 — Summary of the best hits for each query sequences (E-value<1E-40) in fecal samples (0.20 MB PDF) [file pone.0004219.s003.pdf]

Table S2. Summary of the best hits for each query sequences (E-value < 10^{-40}) in fecal samples

#N1		#N2	
Read	Virus	Read	Virus
5	Norovirus Hu/Houston/TCH186/2002/US	1,707	Norovirus Hu/Houston/TCH186/2002/US
		1,646	Norovirus Hu/GII.4/Kobe034/2006/JP
		1,422	Norovirus genogroup 2
		853	Norovirus Hu/NLV/Oxford/B4S6/2002/UK
		328	Norovirus Hu/GII.4/Nijmegen115/2006/NL
		235	Norovirus Hu/NLV/Oxford/B2S16/2002/UK
		233	Norovirus Hu/GII.4/Nijmegen/2007/NL
		210	Norovirus Hu/Norovirus/GII-4/Pecs2967/2007/HUN
		193	Norovirus Hu/GII-4/Narashino/061281/2006/JP
		111	Norovirus Hu/NLV/Oxford/B1S12/2002/UK
		93	Norovirus Hu/GGII.4/5357A/67071/2006/NLD
		62	Norwalk virus
		56	Norovirus Hu/NLV/Dresden174/pUS-NorII/1997/GE
		39	Norovirus Hu/NLV/Oxford/B8S5/2002/UK
		16	Norovirus Env/GGII.4/671/2006/IT
		12	Norovirus Hu/GII.4/MD-2004/2004/US
		11	Norovirus Hu/GII-4/Katori/061492/2006/JP
		10	Norovirus Hu/GII-4/Kashiwa/061256/2006/JP
		9	Norovirus Hu/Sakai/04-179/2005/JP
		9	Norovirus Hu/GII.4/Miyagi/8/2007/JP
		8	Norovirus Hu/GII.4/DenHaag89/2006/NL
		7	Human calicivirus NLV/GII/Langen1061/2002/DE
		6	Norovirus Hu/Norovirus/GII-4/Kapuvar3029/2007/HUN
		6	Norovirus Hu/GGII.4/0029B/69787/2006/NLD
		3	Norwalk-like virus
		2	Norovirus Hu/NLV/Oxford/B4S7/2002/UK
		2	Norovirus Hu/GGII.4/OM2/2002/DNK
		2	Norovirus Hu/GGII.4/5357B/67071/2006/NLD
		2	Norovirus Hu/GGII-4/Benidorm/1707/06/Sp
		2	Norovirus Env/GGII.4/680/2006/IT
		2	Human calcivirus NV/GII/Stockholm/IV6211/2002/SE

Table S2. Summary of the best hits for each query sequences (E-value < 10^{-40}) in fecal samples (continue)

#N3			
Read	Virus	Read	Virus
**813**	**Kyuri green mottle mosaic virus**	**7**	**Citrus tristeza virus**
759	Norovirus Hu/GGII.4/5357A/67071/2006/NLD	6	Norovirus Hu/GII.4/Miyagi/8/2007/JP
583	Norovirus Hu/NLV/Oxford/B2S16/2002/UK	6	Norovirus Hu/GII.4/Miyagi/18/2006/JP
267	Norovirus Hu/Norovirus/GII-4/Pecs2967/2007/HUN	6	Norovirus Hu/Fujian/49747/2006/CHN
231	Norovirus Hu/Norovirus/GII-4/Mosonmagyarovar2594/2006/HUN	5	Norovirus Hu/GII.4/DenHaag89/2006/NL
130	Norovirus Hu/NLV/Oxford/B1S12/2002/UK	4	Norovirus Hu/GII/410/JPN
125	Norwalk virus	3	Norwalk-like virus NLV/Burwash Landing/331/1995/US
120	Norovirus Hu/NLV/Dresden174/pUS-NorII/1997/GE	3	Norovirus Hu/GII-4/C5-159/South Korea
116	Norovirus Hu/GII.4/Nijmegen/2007/NL	3	Human calicivirus Hu/NLV/GII/MD145-12/1987/US
96	Norovirus Hu/Norovirus/GII-4/Kapuvar3029/2007/HUN	**3**	**Enterobacteria phage phiK**
61	Norwalk-like virus	2	Norovirus NVA3
50	Norovirus Hu/GGII.4/OM2/2002/DNK	2	Norovirus Hu/GII-4/Inba/061367/2006/JP
43	Norovirus Env/GGII/670/2006/IT	2	Norovirus Hu/GGII-4/Alzira/1695/06/Sp
39	Norovirus Hu/Sakai/04-179/2005/JP	2	Norovirus Hu/5095/2001/Bra
31	Norovirus Hu/GII-4/Kaiso/060848/2006/JP	2	Norovirus Env/GGII.4/704/2006/IT
30	Norovirus Hu/GII.4/MD-2004/2004/US	2	Human calcivirus NV/GII/Stockholm/IV3355/2002/SE
20	Norovirus Hu/GII.4/Kobe034/2006/JP		
20	Norovirus Hu/GII-4/Kashiwa/061256/2006/JP		
20	Norovirus Hu/Ehime/05-30/2005/JP		
19	Human calicivirus NLV/GII/Langen1061/2002/DE		
17	Norovirus Hu/GII-4/Narashino/061281/2006/JP		
17	Human norovirus Saitama		
13	Norovirus Hu/NLV/Oxford/B4S7/2002/UK		
12	Norovirus isolates		
12	Norovirus Hu/GII.4/Miyagi/5/2006/JP		
11	Norovirus Env/GGII.4/671/2006/IT		
10	Norovirus Hu/Guangzhou/NV-VP2/2006/China		
10	Human calcivirus NV/GII/Stockholm/IV6211/2002/SE		
9	Norovirus Hu/Jilin/50156/2006/CHN		

Table S2. Summary of the best hits for each query sequences (E-value < 10^{-40}) in fecal samples (continue)

#N4		#N5	
Read	Virus	Read	Virus
106	Norovirus Hu/NLV/Oxford/B2S16/2002/UK	**762**	**Pepper mild mottle virus**
57	Norovirus Hu/GII.4/Hunter 532D/04O/AU	188	Norovirus Hu/Chiba/04-1050/2005/JP
50	Norovirus Hu/GII-4/Sanbu/050878/2006/JP	151	Norovirus Hu/Ehime/05-30/2005/JP
49	Norovirus Hu/GGII.4/7366B/63808/NLD	59	Norovirus Hu/GII-4/Funabashi/050601/2005/JP
45	Norovirus Hu/Houston/TCH186/2002/US	50	Norovirus Hu/GII/Shandong/TT147/China
43	Norovirus genogroup 2	38	Norovirus Hu/Sakai/04-179/2005/JP
39	Norovirus Hu/NLV/Oxford/B4S6/2002/UK	34	Norovirus Hu/Guangzhou/NVgz01/CHN
17	Norovirus Hu/NLV/Dresden174/pUS-NorII/1997/GE	27	Norovirus Hu/GII-4/Chiba/040974/2004/JP
14	Norovirus Hu/Chiba/04-899/2004/JP	23	Norovirus Hu/Beijing/CR2905/2004/CHN
**14**	**Human coronavirus HKU1**	20	Norovirus Hu/Guangzhou/NV-VP2/2006/China
11	Norovirus Hu/GII.4/2004/NL	**17**	**Crucifer tobamovirus**
9	Norovirus Hu/NV/Hokkaido/322/2005/JP	6	Norovirus Hu/CHN43172/CC05
7	Norovirus Hu/GII-4/Kimitsu/041440/2005/JP	5	Norovirus Hu/NV/Hokkaido/306/2005/JP
6	Norovirus Hu/NLV/Oxford/B2S11/2002/UK	5	Norovirus Hu/GII-4/Kimitsu/061146/2006/JP
5	Norovirus Hu/Sakai/04-179/2005/JP	2	Norovirus Hu/CHN44898/KM04
4	Norwalk virus	**2**	**Tobacco mosaic virus**
4	Norovirus isolates		
4	Norovirus Hu/Osaka/110/05/JP		
4	Norovirus Hu/GGII.4/Liempde048/2004/NL		
3	Norovirus Hu/GGII.4/Nijmegen083/2004/NL		
**3**	**Phage phiV10**		
**3**	**Human endogenous retrovirus K**		
