# Supplementary material for: Direct Metagenomic Detection of Viral Pathogens in Nasal and Fecal Specimens Using an Unbiased High-Throughput Sequencing Approach
Source: PLoS One. 2009 Jan 19;4(1):e4219. doi: 10.1371/journal.pone.0004219 (PMC2625441; doi:10.1371/journal.pone.0004219)
Supplement: Table S3 — Bias towards TG(CA)-enrichment within the detected norovirus sequences in fecal samples (0.10 MB PDF) [file pone.0004219.s004.pdf]

Table S3. Bias towards TG(CA)-enrichment within the detected norovirus sequences in fecal samples

depth	over 200		below 100	
Rank	Pattern	Z-value[a]	Pattern	Z-value[a]
1	TG	6.7	CT	3.4
2	CA	5.3	AG	3.1
3	GG	2.6	TG	2.2
4	CT	2.1	CA	1.8
5	CC	2.0	TC	1.5

a: The Z-value was calculated using the formulae (1-3), where Z_i is the Z-value of the pattern of two nucleotides *i*, x_{io} is the observed number of the pattern *i*, $\langle x_i \rangle$ is the average number of the pattern *i* in random sample, v_i is its variance, *n* is the number of shuffle trials 100,000, x_{ij} is the number of the pattern *i* at trial *j*.

(1) $Z_i = \left(x_{io} - \langle x_i \rangle\right) / \sqrt{v_i}$

(2) $\langle x_i \rangle = \frac{1}{n} \sum_{j=1}^{n} x_{ij}$

(3) $v_i = \frac{n \sum_{j=1}^{n} x_{ij}^2 - \left(\sum_{j=1}^{n} x_{ij}\right)^2}{n(n-1)}$
